# Swin-Transformer -YOLOv5 for lightweight hot-rolled steel strips surface defect detection algorithm

**DOI:** 10.1371/journal.pone.0292082

**Published:** 2024-01-25

**Authors:** Qiuyan Wang, Haibing Dong, Haoyue Huang

**Affiliations:** 1 School of Electrical and Information Engineering, Hunan Institute of Technology, Hengyang, China; 2 School of Electrical Engineering, University of South China, Hengyang, China; Al Mansour University College-Baghdad-Iraq, IRAQ

## Abstract

An essential industrial application is the examination of surface flaws in hot-rolled steel strips. While automatic visual inspection tools must meet strict real-time performance criteria for inspecting hot-rolled steel strips, their capabilities are constrained by the accuracy and processing speed of the algorithm used to identify defects. To solve the problems of poor detection accuracy, low detection efficiency, and unsuitability of low computing power platforms of the hot-rolled strip surface defect detection algorithm The Swin-Transformer-YOLOv5 model based on the improved one-stage detector is proposed. By employing GhostNet, the model’s lightweight design, and guaranteed detection accuracy are both achieved. The C3 module introduces Swin-Transformer to address the issues of cluttered backdrops of defect photos and easily confused defect categories. With the addition of the CoordAttention module, the model’s capacity to extract defective features is improved, and its performance keeps getting better. The issue of huge differences in different scales and poor detection of small flaws is resolved by employing BiFPN for feature fusion, and the detector’s capacity to adapt to targets of different scales is improved. The experimental results demonstrate that the improved Swin-Transformer-Yolov5 model significantly outperforms the industry-standard target detection algorithms, and the model’s mAP value still improves by 8.39% over the original model while reducing the number of parameters, GFLOPs, and weight by 36.6%, 40.0%, and 34.7%, respectively. The model is better suited for use on low-arithmetic platforms as a result.

## 1 Introduction

Hot-rolled steel is frequently used in sectors like pipeline construction, mechanical construction, and automobile structural steel. Utilizing hot-rolled strip may have inner damage that affects the mechanical properties and corrosion resistance of the strip in addition to surface imperfections, which affect the strip’s appearance. It is crucial to inspect the surface and select the unqualified strips as a result [1–4].

Common automatic vision inspection instruments first identify defective areas from the normal background [5]. The detected defects are then identified and marked. The inspection of hot-rolled steel strips has high requirements for real-time performance, but automatic vision inspection instruments are limited by the accuracy and time efficiency of the algorithm during defect detection. Therefore, the focus of this paper is to reduce the computational overhead of the algorithm while retaining a high accuracy rate. At the same time, the environment at a steel mill site is complex [6]. The suboptimal imaging environment requires detection algorithms that can withstand large intra-class variations and small rapier distances [7]. Eternal continuous image streams require algorithms that balance accuracy with computational complexity [8]. Industry and academia have been trying to figure out how to solve this problem, including hardware upgrades and algorithm optimization. Although the hardware architecture based on server scaling or ASIC acceleration has gained some progress [9–11]. But due to the limitations of Moore’s Law, it is difficult to get a significant breakthrough in hardware in a short period [12]. Therefore, it is very important to obtain performance improvements through algorithmic optimization.

Therefore, this paper presents the Swin-Transformer-YOLOv5 model based on a one-stage detector improvement, which enhances classification accuracy and detection speed of flaws, and the smaller computational size simplifies the deployment of the model on platforms with lower arithmetic capability.

## 2 Literature review

In recent years, with the popularity of artificial intelligence, machine learning has been heavily applied to steel strip surface defect detection. At present, machine learning usually classifies the steel strip surface defect detection task as a binary classification problem, i.e., with or without defects. The current steel strip surface defect detection methods are classified into three main categories: supervised learning, unsupervised learning, and reinforcement learning.

Supervised learning has made rapid progress in steel surface defects compared to unsupervised and reinforcement learning. Ghorai et al. ranked first among all feature classifier combinations by fusing the performance of VVRKFA (classifier) with one-level Haar features [13]. Liu et al. used a two-layer feed-forward neural network to reduce the defect detection task to a binary classification problem, but the large number of parameters of the neural network required a significant computational overhead [14]. Haq M A et al developed CNN based automated weed detection system using UAV imagery. The developed model showed an overall accuracy after rigorous hyperparameter tuning for weed detection, significantly higher than previously reported studies [15]. Cha et al. proposed a deep CNN to detect steel surface defects with higher robustness with reduced computational overhead by tailoring model parameters for convolution and sub-sampling in a convolutional neural network (CNN) [16]. Moreover, the Faster R-CNN de-signed by the team further improves the real-time performance of the detection system [17]. Haq et al proposed Principal Component-based Convolution Neural Network (PCCNN) approach using CNN to detect intrusions, achieving greater precision based on deep learning [18]. YOLO enables CNN-based detection methods to be applied to real-time industrial scenarios by treating the bi-classification task as a regression problem. In the field of single-stage target detection, two classical algorithms, SSD and YOLO, have been widely used for surface defect detection. SSD is simple and efficient, suitable for small target detection, and has a good ability to handle the category imbalance problem. For example, Li et al. proposed a MobileNet-SSD-based method for detecting defects on the sealing surface of containers in filling lines, which simplified the parameters of the detection model [19]. Jawaharlalnehru et al proposed target object detection from unmanned aerial vehicle (uav) images based on improved yolo algorithm. This proposed model can be effectively used for real-time target detection for multi-scale targets with reduced misprediction rate due to its superior accuracy [20]. The YOLO series algorithm uses a multi-scale training and prediction strategy to detect targets of different sizes and shapes. It also employs target classification and regression techniques with faster detection speed and lower computational cost, which is significantly advanced in the field of strip steel surface defect detection. For example, Li et al applied an improved YOLO network to the detection of surface defects in cold-rolled strip steel and achieved high accuracy and detection precision [21]. However, it is difficult to detect defects with an area of less than 10 square millimeters. Kou et al. developed an end-to-end defect detection model based on YOLO-V3 [22]. Although the model’s FPS is higher than ours, our mAP is higher than theirs while the model is lighter and more suitable for deployment on mobile platforms. Li et al proposed an improved algorithm based on YOLOv4 [23].

Transformer has achieved great success in natural language processing in recent years [24–26]. With the advent of Vision Transformer (ViT), Transformer has surpassed most CNN-based approaches on various computer vision tasks. Vision Transformer works by dividing the input image into two-dimensional Patches, which are then mapped into one-dimensional vector sequences by a trainable linear mapping matrix. Finally, the one-dimensional vector sequence is fed into the standard Transformer architecture to learn the available feature representations. Also, Transformer has some applications in the field of target detection. For example, Zhang et al. proposed utilizing Vision Transformer with the ability to model remote dependencies using a self-attentive mechanism for image recognition [27]. However, it is found that ViT maintains the same feature resolution in all feature extraction stages and computes the dependencies among the pixels within the whole image, which leads to the problem that ViT cannot extract multi-level representation of the image and has high computational complexity. Therefore, an attempt is made to explore the Swin Transformer [28] as the basis for designing the network by introducing the local idea and hierarchical structure into the Transformer to solve the above problems.

## 3 Proposed methods

**Fig 1:**
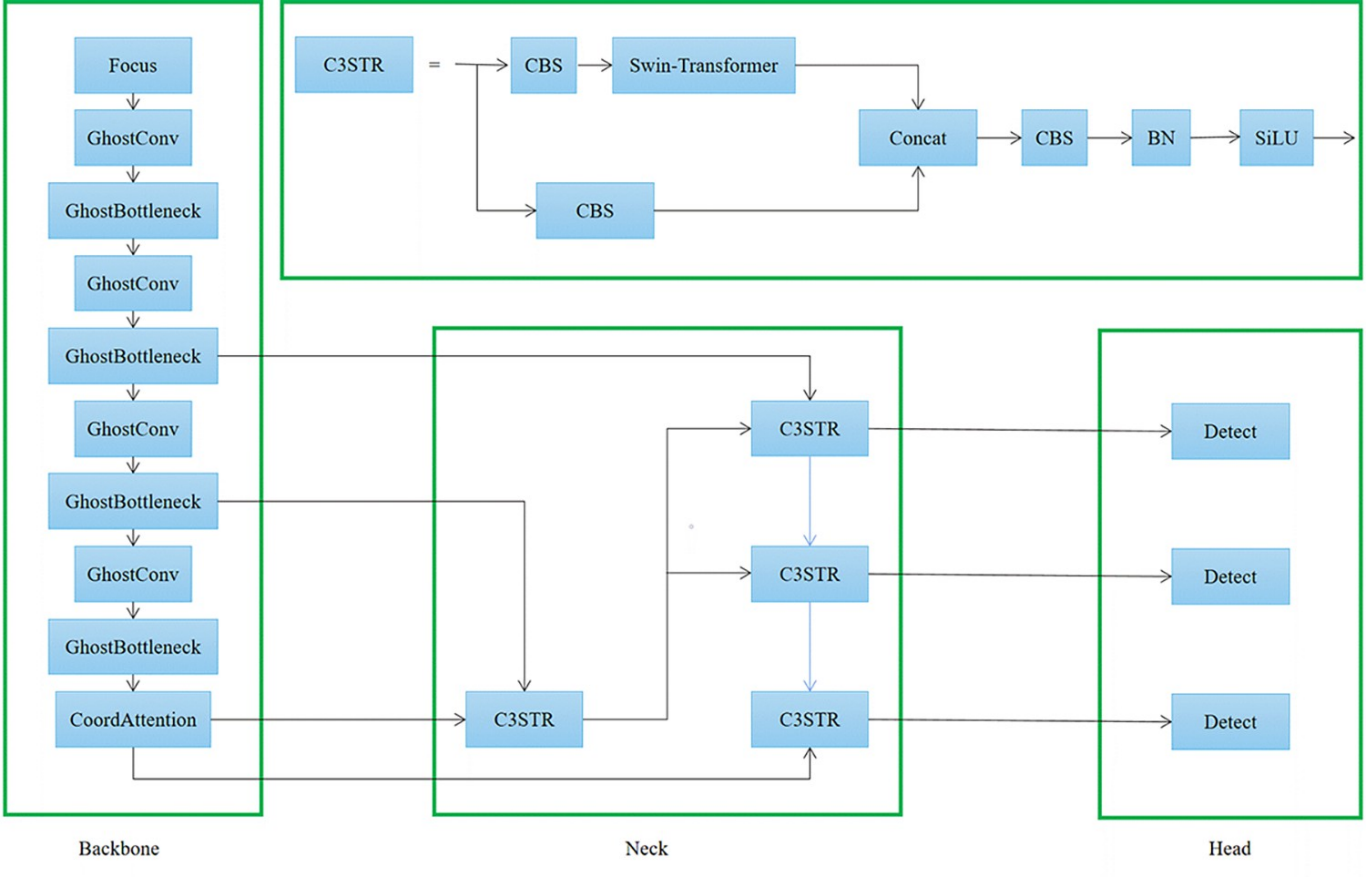
Network structure of Swin-Transformer-YOLOv5.

The YOLOv5s algorithm is the lightest version of YOLOv5 (version 5.0) [29]. The algorithm for detecting surface imperfections in hot-rolled steel strips is developed using migration learning on the YOLOv5s pre-trained model [30]. The algorithm is made up of the following parts: Input, Backbone, Neck, and Head. The input is a 640 × 640 × 3 image, which is then processed using Mosaic data enhancement, adaptive image filling, and preprocessing operations. The Backbone network is a high-performance classifier network that is used to extract generic features from the target. Its primary structural components are a slicing structure (Focus), a convolution module (Conv), a bottleneck (C3), and pyramidal pooling (SPP). Path aggregation and feature pyramid (FPN) are used in the Neck network structure (PAN). The head is mainly used for the final detection part, which applies anchor frames on the feature map and generates the final output volume with class probabilities, object scores, and enclosing frames. By swapping out the Conv in the Backbone and Neck of the YOLOv5s with GhostConv and the C3 module in the Backbone with the GhostBottleneck module, the Swin-Transformer-YOLOv5 algorithm proposed in this paper makes the model lighter. The Swin-Transformer is fused into the C3 module, and the C3STR module is utilized in place of the Neck C3 module. The CoordAttention module is positioned in the last layer of the backbone network, and the method of using PANet feature fusion in YOLOv5 is modified to using BiFPN for feature fusion. This leads to a lightweight design and better overall model performance by enabling the network precision to be increased and more features to be merged with little to no additional computational overhead. Fig 1 depicts the Swin-Transformer-YOLOv5 network structure.

### 3.1 Replace the Yolo5 Backbone and Neck with GhostNet

**Fig 2:**
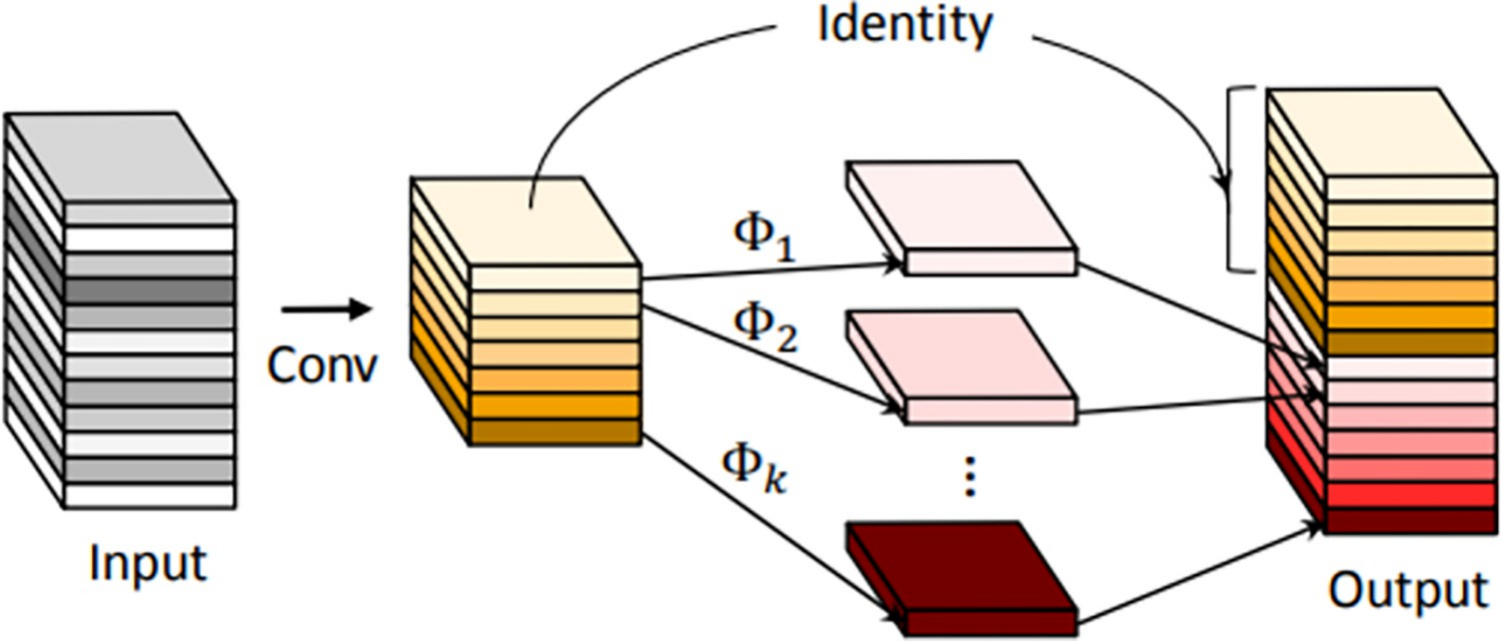
The structure of the Ghost module.

Rich redundant features in the YOLOv5s model increase the generalization of the model but also create the issue of computational hit; therefore, the light weight of the model is achieved by introducing GhostNet in order to ensure the thorough interpretation of the input by the model [31]. Fig 2 depicts the Ghost module’s structural layout.

The specific process of GhostNet is

$$Y^{\prime }=X*f^{\prime }$$ (1)

$$y_{ij}=\Phi_{ij}(y_{i}^{\prime }),i\in 1,2,\ldots ,m,j\in 1,2,\ldots ,s$$ (2)

The first step is the convolution operation, *X* is the input feature map, *Y*′ is the output m feature maps, and *f*′ is the convolution kernel of size *K*×*K*. The second operation is the linear operation *Φ*_*ij*_ for each feature map $y_{i}^{\prime }$ in *Y*′, and finally *n* = *ms* output feature maps *Y* = [*Y*_11_, *Y*_12_,…,*Y*_*ms*_] are obtained, and after calculation, it can be concluded that the ordinary convolution operation is about s times of Ghost module.

**Fig 3:**
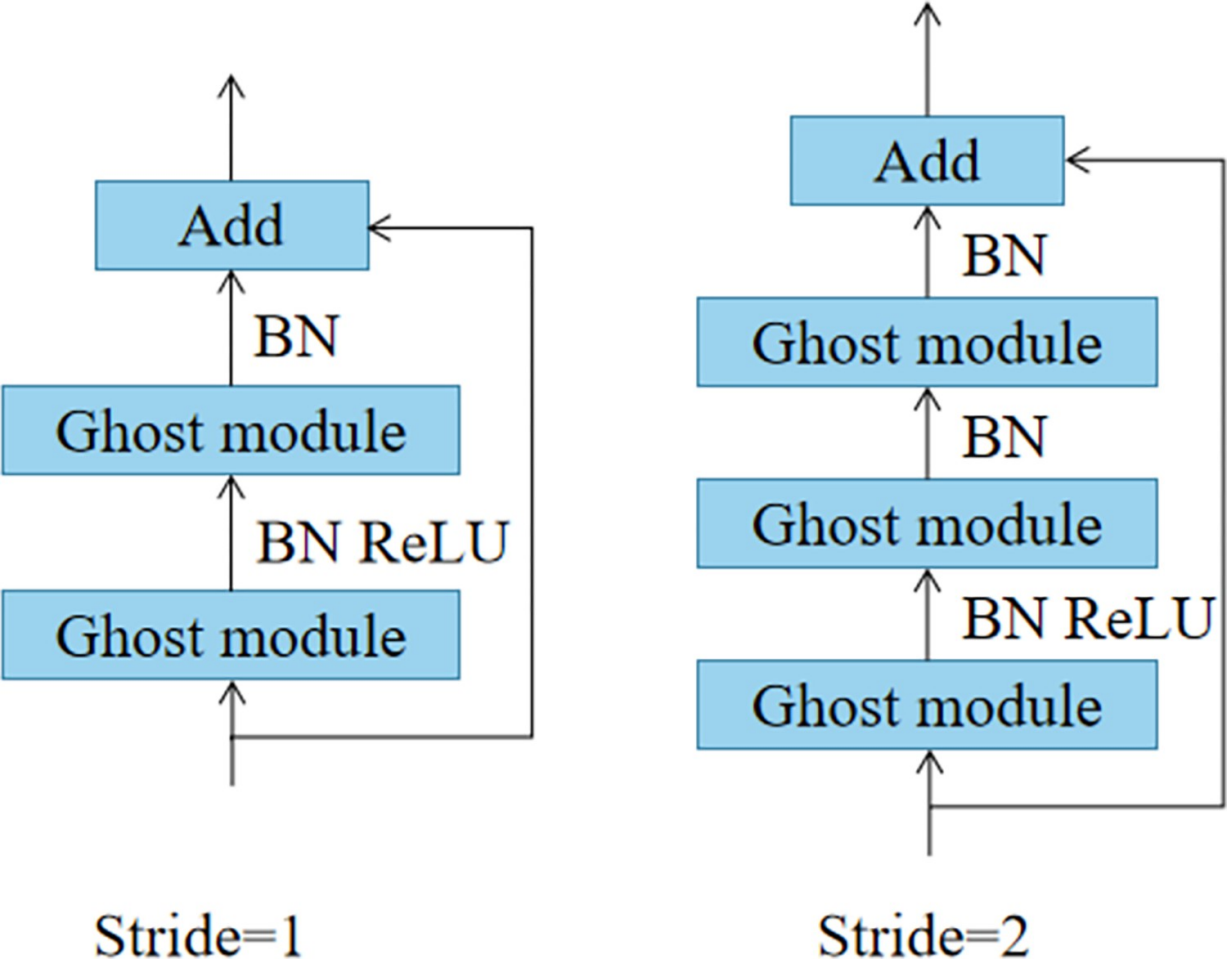
The structure of the GhostBottleneck module.

The GhostBottleneck module, which consists of two Ghost Modules and replaces the features provided by regular convolution with features produced by a straightforward linear transformation, significantly reduces the model complexity. When Stride = 1, the backbone is made up of two Ghost Modules (GMs), where the first GM increases the number of channels and the second GM decreases the number of channels to match the number of input channels, and the remaining edge component is identical to ResNet. Since S = 1, the input feature layer’s height and width are not compressed, and its purpose is to increase the network’s depth. When Stride is 2, a Deepwise convolution with a stride of 2 is added in the backbone region between the two GMs, which can condense the feature map’s height and breadth to make it only half as large as the input. To guarantee that the Add operation may be aligned, a deep separable convolution with a step size of 2 × 2 and a standard convolution of 1 × 1 are additionally added to the residual edge part. The purpose of S = 2 is to modify the geometry of the input feature layer by compressing the height and width of the input feature layer. Fig 3 depicts the GhostBottleneck module’s structural layout.

The Conv in the Backbone and Neck of the YOLOv5s is replaced with GhostConv to make the entire model lighter, and the C3 module in the Backbone is replaced with GhostBottleneck to speed up computation.

### 3.2 C3 module incorporating Swin-Transformer

**Fig 4:**
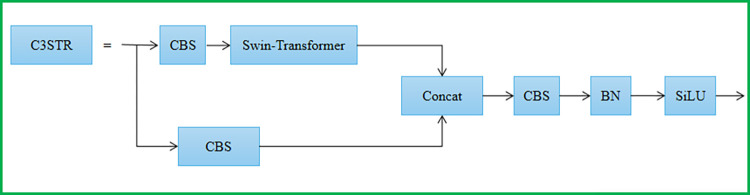
The structure of the C3STR.

After several convolution processes, the high-level feature map loses the majority of the target feature information that a small target in the image should have as the network structure is deepened. Therefore, in the feature fusion part, we borrow the idea of the Swin-Transformer and embed it into the C3 module, replacing the four C3 models in Neck [32]. By introducing some discrete parameters of the Transformer and enhancing the semantic information and feature representation of small targets using the help of the window self-attention module. Fig 4 depicts the C3STR with a Swin-Transformer.

Swin-Transformer features are learned by moving the window. Moving the window improves efficiency because self-attention is computed within the window, so as long as the window size remains constant, the computational complexity of self-attention remains constant, and the total computational complexity is a linear multiple of the image size, reducing the length and computational complexity of the sequence. The capacity to interact between two neighboring windows is enabled by the shifting operation at the same time, creating a cross-window connection between the upper and lower levels and thus enabling global modeling.

The calculation process of the multi-headed self-attentive mechanism is as follows:

$$Attention(Q,K,V)=SoftMax(\frac{QK^{T}}{\sqrt{d}}+B)V$$ (3)

Attention denotes attention and SoftMax denotes the normalization function. Where *Q*, *K*, and *V* are query, key, and value matrices; *d* is the query/key dimension; and *B* is a smaller-sized bias matrix. Introducing *B* can effect significant improvement.

The C3STR module controls the computational region in each window by dividing the local window to achieve cross-window information interaction, lowering the sequence length and computational complexity as compared to the Multi-head Self-Attention in the conventional Transformer.

### 3.3 CoordAttention module

**Fig 5:**
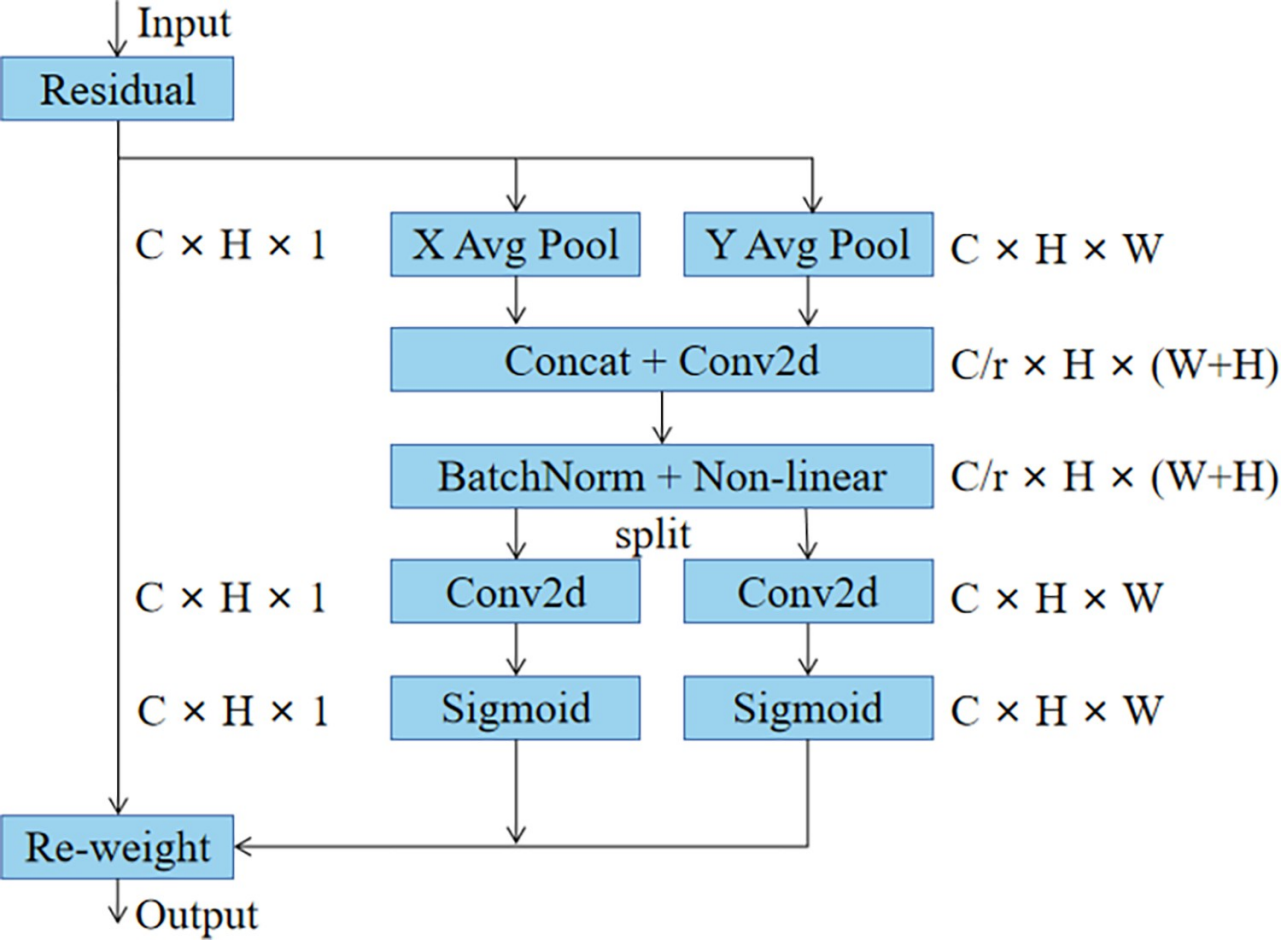
The structure of the CoordAttention module.

The application of attention mechanisms is hampered by the fact that lightweight networks cannot afford the higher computational overhead [33]. At the same time, the attention mechanism improves the model’s accuracy while ignoring location information, which is critical to the network. CoordAttention, as an attention mechanism designed for lightweight networks, is a good solution to the above problem [34]. Coordinate information embedding and coordinate attention generation are the two components of Coor-dAttention. The global pooling is transformed into a pair of one-dimensional feature encodings in information embedding, allowing the CoordAttention module to capture remote spatial interactions with location information. Long-range dependencies are captured along one spatial direction during attention generation, while precise location information is retained along the other. The module acquires a good global sensory field and encodes precise location information, which improves the module’s ability to capture regions of interest. The module is simple, flexible, and efficient, and its placement at the backbone network’s final layer can improve network accuracy while incurring little computational overhead. Fig 5 depicts the CoordAttention module’s structure.

### 3.4 Replace PANet with BiFPN

**Fig 6:**
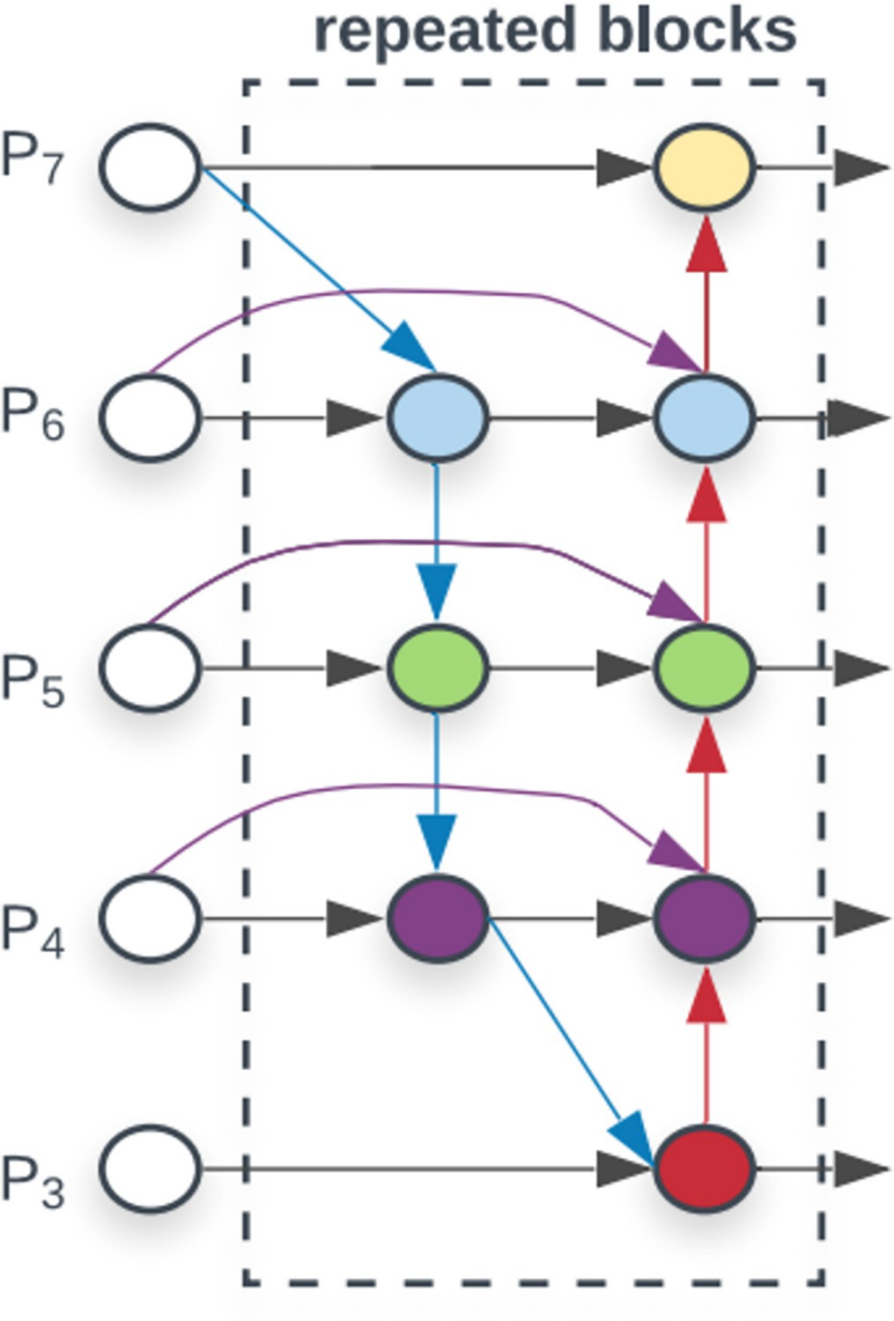
The structure of the BiFPN.

The semantics of the features change from low latitude to high dimensional as the network layers deepen. Each layer of the network will cause a certain degree of feature loss, and the semantic information can be enriched by fusing the features of different layers. The feature fusion method of YOLOv5 using PANet is changed in this paper to feature fusion using BiFPN to construct a feature pyramid, and the semantic features extracted from the backbone network are fused top-down using efficient bi-directional cross-scale connectivity and weighted feature map fusion. The shallow network can contain clearer location information due to larger resolution, and the deep network can contain more high-dimensional semantic information due to the large sensory field [35, 36]. More features can be fused without increasing the cost by adding lateral connections between the original input and output nodes of the same feature. The improved YOLOv5s network will extract features at various scales from the backbone network’s GhostBottleneck2 and GhostBottleneck3 layers, which will then be fed into the BiFPN network. Fig 6 depicts the BiFPN structure diagram.

## 4 Experiments

### 4.1 Datasets

**Fig 7:**
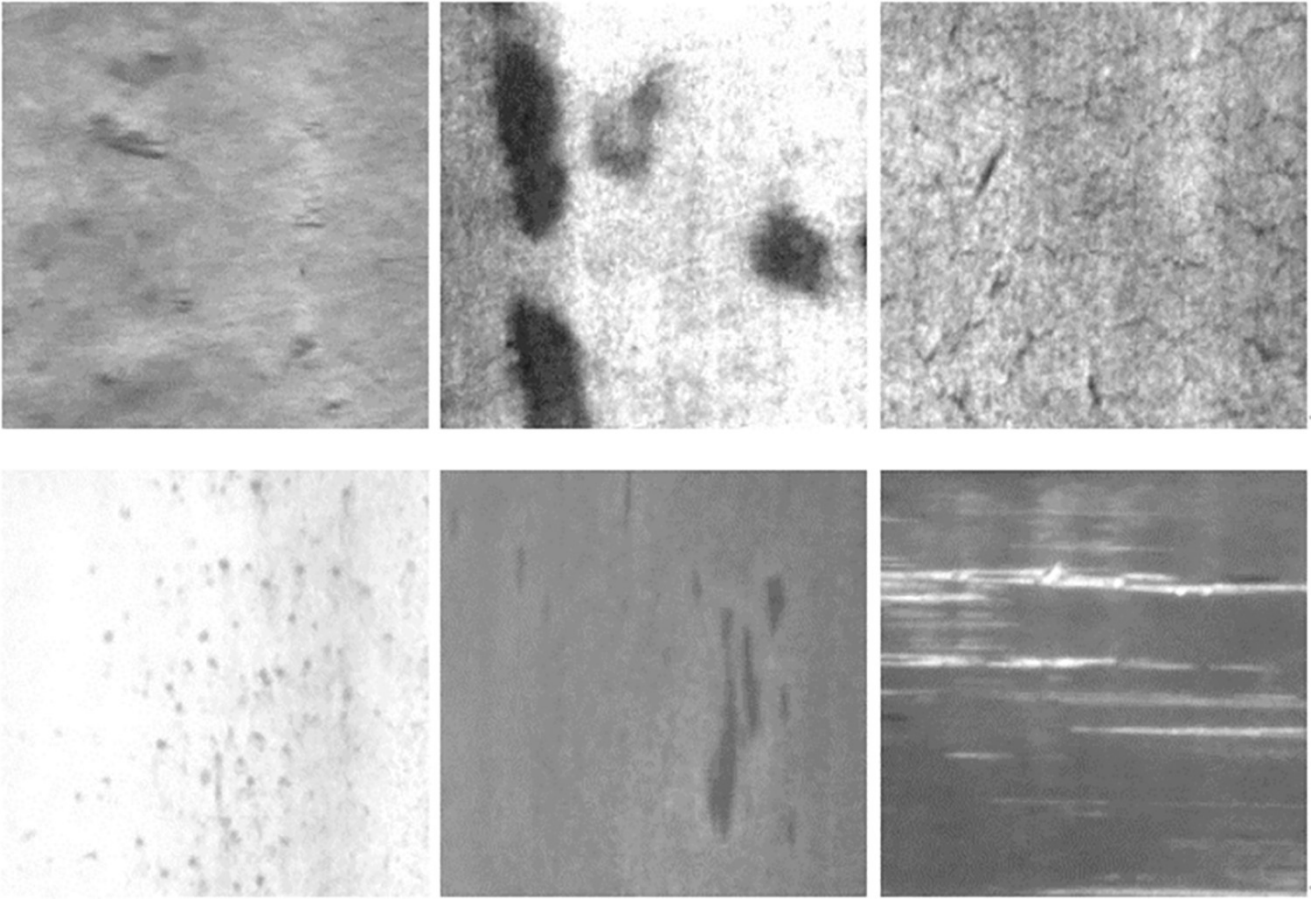
The six typical defects.

The dataset used for the experiments is a dataset of hot-rolled strip surface defects published by Northeastern University: the dataset includes six typical hot-rolled strip surface defects, which are rolled-in scale, patches, crazing, pitted surface, inclusion, and scratches [37]. The database contains a total of 1800 grayscale images, with 300 images per category. Using LabelImg software, the dataset was divided into training datasets, validation datasets, and test datasets according to 7:2:1. Fig 7 depicts the six typical defects.

**Fig 8:**
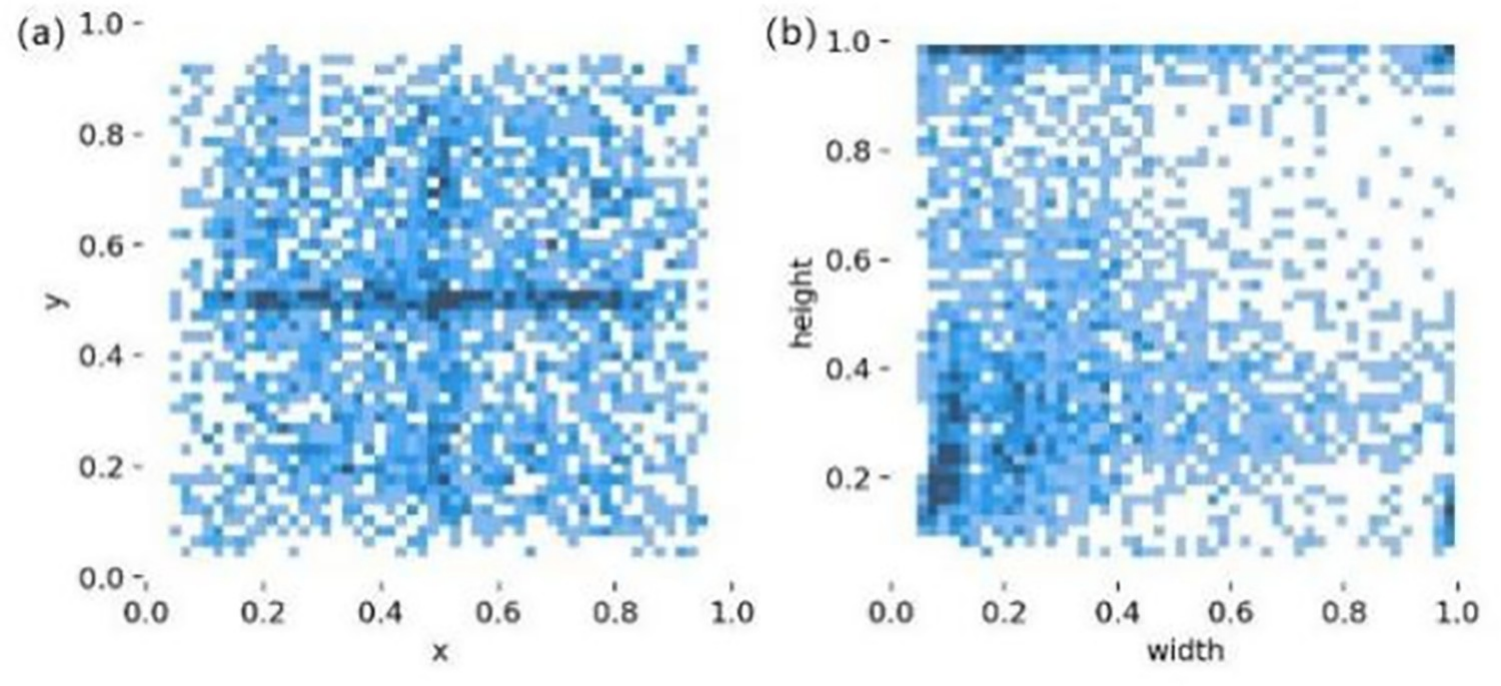
(a) Analysis of the center point of the callout box; (b) Analysis of the size of the callout box.

The label box center point coordinates (x, y), width, and height of the hot-rolled steel strip surface defect dataset are normalized. Fig 8(A) shows the distribution of the center coordinates of the label. From the figure, it can be seen that the center coordinates are distributed almost anywhere in the image, which indicates that the defects are uniformly distributed anywhere in the image. Fig 8(B) shows the height and width distribution of the label, and it can be seen that the dark part is mainly distributed in the lower left corner of the image, although it is also distributed in all other positions. This indicates that the defects to be detected are mainly small and medium targets, although there are also some large targets.

### 4.2 Training details

The experimental environment in this paper is based on the Windows 10 operating system, with AMD R7 4800H CPU, NVIDIA GeForce RTX2060 GPU with 6 GB video memory, 16 GB RAM, PyTorch 1.12.1 as the deep learning framework, Python version 3.8.13, and CUDA version 11.7.

This training is performed using migration learning by loading pre-trained weight parameters on the hot-rolled strip surface defect dataset and continuing the training 300 times. The input image size is 640 × 640, the Batch_size is set to 16, the initial learning rate is 0.01, the momentum is 0.937, the weight decay is set to 0.0005, and the Epoch is set to 300. The model learning rate will gradually become larger during the first three Epoch training runs; after three Epochs, the learning rate will become 0.01, and with each Epoch iteration, the learning rate will gradually become smaller to prevent the model from overfitting while ensuring the deep temperature of the model.

## 5 Results and discussions

### 5.1 Experimental results and ablation study

Table 1 shows the specific parameters of each model and the results of the ablation experiments. Analysis of the experimental results shows that our model has a 36.6% decrease in parameters, a 40.0% decrease in GFLOPs, a 34.7% decrease in weight, and an 8.39% increase in mAP compared to the YOLOv5s model. This is primarily due to the fact that GhostNet generates features using simple linear transformation rather than normal convolution, which significantly reduces model complexity while also reducing model accuracy to some extent. Although there is a small increase in parameters, GFLOPs, and weight when the CoordAttention module is added after Backbone, the mAP improves by 3.3%, demonstrating that the addition of CoordAttention improves the model’s ability to extract defective features and significantly increases the overall model accuracy improvement. Changing the PANet in the Neck of the YOLOv5s to BiFPN improves the mAP by 2.2% compared to the YOLOv5s model with a small reduction in parameters and GFLOPs and a slight increase in weight. This suggests that by fusing features of different scales, BiFPN improves the detector’s ability to adjust to targets of different scales, as well as the problem of poor detection ability for defects with large scale variations and small defects. By replacing the C3 module in Yolov5’s Neck with the C3STR module incorporating the Swin-Transformer, the mAP improved by 5.9% with a slight increase in parameters, GFLOPs, and weight. This suggests that the addition of the Swin-Transformer improves the problem of cluttered backgrounds of defect images and easy defect type confusion.

**Table 1:** Specific parameters of each model and ablation experiments.

Method	Parameters(M)	GFLOPs	Weight(M)	mAP(%)	FPS
YOLOv5s(baseline)	7.08	16.5	14.4	69.1	52.1
+ GhostNet	4.17	9.3	8.7	69.5	50.7
+ C3STR	7.29	17.0	15.0	73.2	47.5
+ CoordAtt	7.46	17.0	15.2	71.4	51.2
+BiFPN	7.00	16.4	16.3	70.6	51.4
+CoordAtt+BiFPN	7.18	16.6	14.6	73.0	50.6
+ GhostNet+ C3STR	4.37	9.8	9.2	72.5	45.9
+GhostNet+CoordAtt+BiFPN	4.28	9.4	8.9	72.6	48.3
Swin-Transformer-YOLOV5	4.49	9.9	9.4	74.9	43.2

The mAP of YOLOv5s and our model for detecting surface defects in six types of hot rolled strips are shown in Table 2. Table 2 shows that our model improved the mAP of rolled-in scale, patches, crazing, pitted surface, inclusion, and scratches by 21.0%, 6.9%, 12.7%, 10.9%, 5.5%, and 1.25% when compared to the YOLOv5s model. For YOLOv5s poor detection results of rolled-in scale, crazing has a large improvement.

**Table 2:** The effect of Swin-Transformer-YOLOV5 and YOLOV5S on 6 types of defects detection.

Method	rolled-in scale	patches	crazing	pitted surface	inclusion	scratches
YOLOv5s	49.9	85.3	35.8	77.4	78.2	88.0
OURS	60.4	91.2	40.3	85.8	82.5	89.1

According to the above analysis, the addition of GhostNet effectively reduces the model’s parameters, GFLOPs, and Weight. The addition of Swin-Transformer, BiFPN, and CoordAttention significantly improves the model’s mAP.

**Fig 9:**
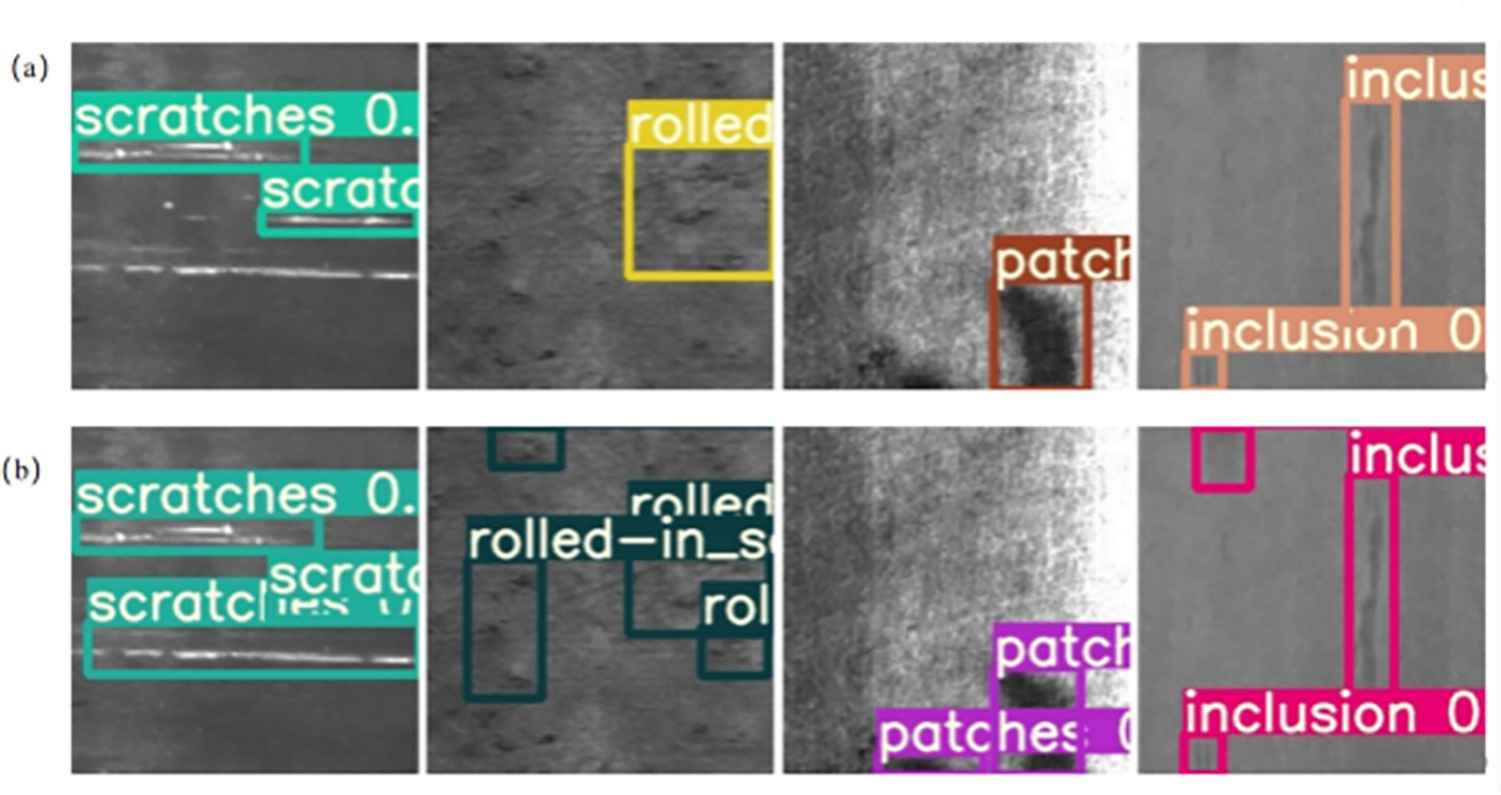
(a) Detection effect of YOLOv5s; (b) Detection effect of Swin-Transformer-YOLOv5.

Fig 9(A) and 9(B) show some of the detection results of the YOLOv5s model and our model. According to the comparison plots, YOLOv5s has a problem with missed detection, especially for small targets, whereas our model improves the detection rate in this regard.

### 5.2 Comparison of different algorithms

In this paper, SSD, Faster R-CNN, YOLOv3, YOLOv4, Retina-Net, and Swin-Transformer-YOLOv5 were selected for performance comparison, and the test results were derived from the same dataset. From Table 3, we can find that the mAP of our model was much higher than that of its counterparts, YOLOv3 and YOLOv4, which may be due to the poorer capture of small defects by YOLOv3. Meanwhile, mAP was significantly ahead of SSD and Faster R-CNN models and slightly ahead of Retina-Net, due to the fact that our model combines different levels of features and background effects of the images. However, extracting more complex features also had a negative impact in terms of FPS. In general, the performance of Swin-Transformer-YOLOv5 in this paper was excellent. For the six hot-rolled strip surface defects, Swin-Transformer-YOLOv5 outperformed the five models except Retina-Net on all six defects. Compared with the Retina-Net model, only two categories of crazing and inclusion lag, which indicate that there is room for further optimization of the model. In terms of detection speed, Swin-Transformer-YOLOv5 achieved an FPS of 43.2, which indicates that the model can satisfy the real-time detection of surface defects in hot-rolled strips.

**Table 3:** Performance of different algorithms.

Types	SSD	Faster-RCNN	YOLOv3	YOLOv4	YOLOv5	Retina-Net	OURS
rolled-in scale	70.8	54.5	30.8	38.7	49.9	43.5	60.4
Patches	61.9	75.3	82.5	87.9	85.3	91.1	91.2
Crazing	30.3	25.0	21.4	15.6	35.8	45.9	40.3
pitted surface	39.1	73.6	77.0	68.6	77.4	74.7	85.8
Inclusion	50.0	65.1	62.1	72.3	78.2	84.2	82.5
Scratches	51.1	81.1	83.2	82.0	88.0	81.6	89.1
mAP	51.0	62.4	58.2	61.7	69.1	70.2	74.9
FPS	31.5	23.8	60.7	44.7	52.1	47.8	43.2

**Fig 10:**
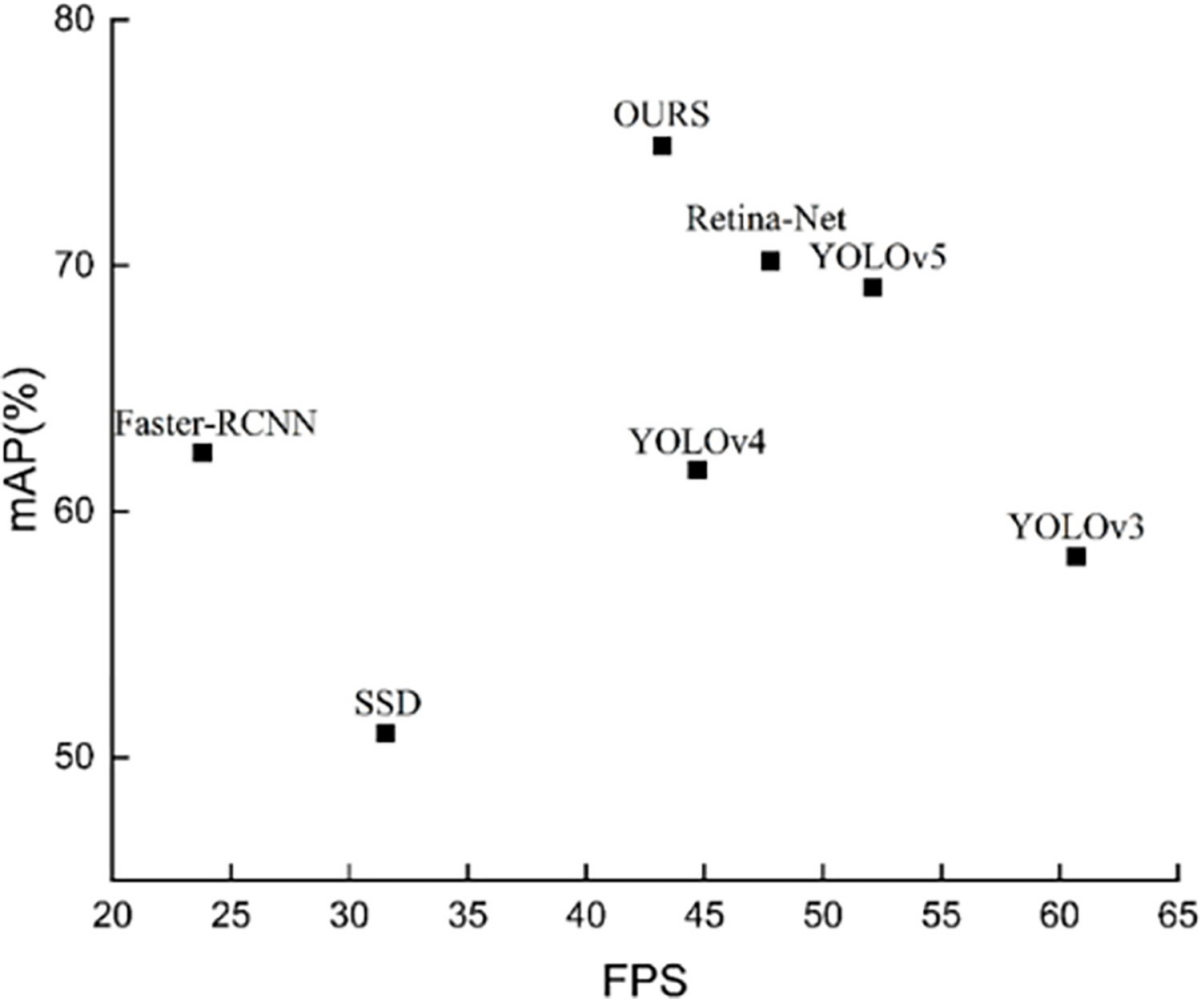
Performance evaluation matrix for different algorithms.

The performance evaluation matrix of different algorithms is shown in Fig 10. Compared with Faster-RCNN and SSD, our model had a huge advantage in both mAP and FPS dimensions. YOLOv4 and Retina-Net with similar processing speed were lower than our model in mAP. YOLOv3, which has a faster processing speed, lagged behind our model substantially in mAP. This shows that the performance of Swin-Transformer-YOLOv5 in this paper was excellent.

## 6 Conclusions

In this paper, an algorithm named Swin-Transformer-YOLOv5 is designed for detect surface defects on hot rolled strip steel. Swin-Transformer, GhostNet, CoordAttention, and BiFPN are some of the modern computer vision techniques that are combined in Swin-Transformer-YOLOv5. To address the issue of a large number of parameters and computation, GhostConv and GhostBottleneck were proposed to be utilized in Backbone, and GhostConv was used in Neck to keep the model lightweight. The improved YOLOv5 network is more suited for real-world industrial applications since it features a lighter network concept and lower demanding hardware. It was proposed that the C3 module be used in conjunction with the Swin-Transformer at Neck to address the issues of cluttered defect image backdrops and simple defect type confusion. The usage of BiFPN rather than PANet was suggested to increase the detector’s ability to adjust to targets of varied scales by fusing characteristics of multiple scales, which would address the difficulties of high variance in defect scales and poor detection of minor defects. The model’s capacity to extract defective features was enhanced by the CoordAttention module. The improved model obtained a 74.9% mAP with a 36.6% decrease in parameters, a 40.0% decrease in GFLOPs, a 34.7% decrease in weight, an 8.39% improvement over the baseline, and an FPS of 43.2 when tested on the dataset. The experimental results show that the improved network can achieve better detection with fewer parameters while retaining the potential for real-time monitoring. There is still potential for development in terms of detection speed and efficiency even though the enhanced model performs better than the majority of the current target detection methods. In the next study, the model will introduce a richer dataset to strengthen its generalization capability and improve the model’s real-time monitoring capability. Meanwhile, the network structure is further optimized to enhance the extraction of features and improve the detection speed and accuracy of the network. Study how to deploy the model on mobile, and refine and improve the model in real defect detection applications.
